# Consecutive Microsurgical Cases Performed by Single Surgeon at a Canadian Tertiary Care Center: A Retrospective Review

**DOI:** 10.1177/22925503241234934

**Published:** 2024-03-01

**Authors:** Andrew T. Chen, Jessica Gormley, Oluwatobi Olaiya, Sophocles H. Voineskos, Christopher J. Coroneos, Ronen Avram

**Affiliations:** 1 Division of Plastic Surgery, Department of Surgery, 3710 McMaster University, Hamilton, ON, Canada; 2 Division of Plastic, Reconstruction and Aesthetic Surgery, Department of Surgery, University of Toronto, Toronto, ON, Canada; 3 Department of Health Research Methods, Evidence and Impact, 3710 McMaster University, Hamilton, ON, Canada

**Keywords:** free flap, reconstruction, surgeon career, microsurgery, Mots-clés, Lambeau libre, reconstruction, carrière de chirurgien, microchirurgie

## Abstract

**Background:** The practice of a microsurgeon evolves over time with experience, changes in clinical interest, and practice setting. Previous reports suggest that complication rates may be influenced by years of practice. The aim of this study was to analyze consecutive microsurgical cases performed by a single surgeon during the first half of their career in a broad microsurgical practice at a Canadian academic tertiary care center. **Methods:** A retrospective chart review of all free tissue transfers between 2007 and 2020 completed by a single academic plastic surgeon was performed. The primary outcomes were the odds of postoperative complications and free flap failure. The secondary outcomes included the annual number and type of free flap cases over time. **Results:** The surgeon performed 795 free flaps on 537 patients. There were a total of 131 postoperative complications. There was no statistically significant association between the years in practice and odds of a complication. Overall, there were 26 flap failures, yielding a 96.7% success rate. There was no association between flap failure and years in practice. The most common type of reconstruction was breast, followed by lower extremity and head and neck. There was a significant change in the type of reconstruction cases over time (*P* < 0.005). **Conclusion:** The type and volume of free flap reconstruction conducted by the surgeon has changed over time. In this single surgeon career, surgical experience did not have a significant impact on free flap complications. At our center, free tissue transfer remains a valuable tool for reconstruction in both low and high-risk patients.

## Résumé

**Contexte :** La pratique d’un chirurgien spécialisé en microchirurgie évolue au fil du temps et de l’expérience, change en fonction des centres d’intérêt cliniques et du cadre d’exercice. Des rapports antérieurs suggèrent que les taux de complications peuvent être influencés par les années d’exercice. Le but de cette étude était d’analyser des cas consécutifs de microchirurgie réalisés par un seul chirurgien pendant la première moitié de sa carrière dans un grand centre de pratique microchirurgicale d’un centre de soins tertiaire universitaire au Canada. **Méthodes :** Une revue rétrospective a été menée sur les dossiers de tous les transferts de tissus libres réalisés entre 2007 et 2020 par un seul chirurgien plasticien universitaire. Les principaux critères d’évaluation étaient les chances de complications postopératoires et d’échec des lambeaux libres. Les critères d’évaluation secondaires ont inclus le nombre et le type de cas de lambeaux libres pratiqués annuellement au fil du temps. **Résultats :** Le chirurgien a pratiqué 795 lambeaux libres sur 537 patients. Il y a eu un total de 131 complications postopératoires. Il n’y avait pas d’association statistiquement significative entre le nombre d’années d’exercice et les chances de complications. Globalement, il y a eu 26 échecs de lambeaux, correspondant à un taux de succès de 96,7%. Il n’y a pas eu d’association entre l’échec des lambeaux et les années d’exercice. Le type de reconstruction le plus fréquent était la reconstruction du sein, suivie par celles des membres inférieurs et de la tête et du cou. Il y a eu un changement significatif du type des cas de reconstructions au fil du temps (*P *< 0005). **Conclusion :** Le type et le volume des reconstructions avec lambeaux libres réalisés par le chirurgien ont changé au fil du temps. Dans cette unique carrière de chirurgien, l’expérience chirurgicale n’a pas joué un rôle significatif sur les complications concernant les lambeaux libres. Dans notre centre, le transfert de tissus libres reste un outil de valeur pour la reconstruction à la fois chez des patients à risque élevé et chez des patients à risque faible.

## Introduction

Throughout the career of a surgeon, the volume, type, and complexity of procedures they perform can vary. This is dependent on several factors such as gained experience and expertise, personal preference, and changing approaches in the field. In addition, their case profile is heavily influenced by other surgeons with similar clinical interests within the same catchment area.

Free tissue transfer remains at the top of the reconstructive ladder for management of complex defects. It has become an increasingly reliable technique in the past few decades for head and neck, trunk, extremity, and breast reconstruction.^1^ Despite the reported success rates being upward of 95%, the impact of flap failure and other major complications on the proportion of patients experiencing these outcomes remains significant.^2,3^ Previous reports demonstrate a 5% relative decrease in odds of major postoperative complications for every 10 year increase in surgeon's age, suggesting that additional experience improves patient's outcomes.^4^ Specifically related to microsurgery, years in practice has a positive impact on likelihood of free flap success.^5^ Beyond success rates, no previous study has analyzed the trajectory of a microsurgeon's career, and adjusted outcomes for caseload.

In addition to microvascular skill and surgeon experience, there are a number of patient and flap-specific factors that influence success in free flap reconstruction.^6–8^ These include co-morbidities such as hypertension, peripheral vascular disease, diabetes, previous radiation, and smoking history.^7,8^ A recent systematic review by Qian et al demonstrated extremity defects and single perforator flaps were independent risk factors for complications following free flap reconstruction.^6^ Although it is important to consider factors that impact free flap success for patient counseling and clinical decision making, in the setting of traumatic or oncologic reconstruction, operating on high-risk patients remains unavoidable at tertiary centers. For this reason, there has been a recent shift in the literature to look at consecutive cases of free tissue transfer procedures, to better delineate the true complication risk profile across both low- and high-risk patients.

The aim of this study was to analyze consecutive microsurgical cases performed by a single surgeon during a 13-year career at affiliated Canadian tertiary care centers. This represents the first half of their career. We will determine the association years in practice has on postoperative complications and free flap failure. Secondarily, we will analyze how their practice has evolved through time and highlight changes in both volume and reconstructive category.

## Methods

### Patient Population

A retrospective chart review was performed on all consecutive patients undergoing free flap reconstruction by a single surgeon between February 2007 and December 2020 at an academic tertiary care center. The surgeon was primarily responsible for breast reconstruction at a regional cancer center and limb reconstruction at a level one trauma center for the majority of the period. The cohort was created with patients identified using procedural billing codes for preparation of the microvascular recipient site (Appendix A). Patients with inadequate documentation to confirm their eligibility were excluded. In total, there were <5% of patients excluded. This study was approved by the institution's research ethic's board and reported in accordance with the STROBE statement guidelines.^9^

### Data Collection

Once eligibility was confirmed, data was extracted from the patient's electronic medical record. The data collected included patient demographics (age, gender, BMI, smoking history, diabetes, and previous radiation), reconstructive category (breast, head and neck, lower extremity, upper extremity, and abdominal wall), primary diagnosis, procedure details (date of surgery, donor flap type, concomitant operations, bilateral or unilateral where applicable), and complications. Complications included partial necrosis, flap re-exploration, donor site complications (eg abdominal hernia, wound dehiscence), deep vein thrombosis (DVT), pulmonary embolism (PE), hematoma/seroma requiring intervention, infection, and flap failure. While traumatic digital replantations and revascularizations were performed by the surgeon, they were excluded from flap outcomes. Data was extracted independently by one of the authors into a Microsoft Excel spreadsheet.

### Outcome Measures

The primary outcome of this study included complications and free flap failures, and the association with year in practice. The secondary outcomes included the volume and type (breast, head and neck, lower extremity, upper extremity, and abdominal wall) of microsurgical procedures completed per year.

### Statistical Analysis

We summarized categorical data reporting frequencies and percentages, and continuous data reporting the mean and standard deviation (SD). Pearson's chi-squared test was used to evaluate the association between the type of reconstruction and year of practice. Univariable and multivariable logistic regression was used to evaluate the relationship between years in practice and complications (including free flap failure). In the adjusted logistic regression models, we included the variables number of cases per year and type of reconstruction cases as there is an expected confounding relationship between these factors and number of complications and flap failures. The crude odds ratios (OR) and adjusted odds ratios (aOR), corresponding 95% confidence intervals (CI), and *P*-values are reported. The level of statistical significance was set at α = 0.05. Statistical analyses were conducted using R version 4.0.4 (R Foundation for Statistical Computing, Vienna, Austria) or STATA IC, version 16 (StataCorp, LLC, College Station, Texas).

## Results

### Patient Demographics

The cohort consisted of 537 patients, who underwent 552 reconstructive operations, and 795 free flaps (Table 1). The mean age was 53 years (range 15–93, SD 13), 79% were female, and the average BMI was 30 kg/m^2^ (range 18.4–51.3, SD 6); 14% of patients were active smokers, 25% had received previous radiation therapy, and only 9% of patients were diabetic. Additional demographic details are presented in Tables 1 and 2.

**Table 1.** Patient Demographics.

Patient characteristics (N = 537)	
Age, mean (SD)	53 (13)
BMI, mean (SD)	30 (6)
Gender, n (%)	
Male	115 (21)
Female	422 (79)
Smoking status, n (%)	
Non smoker	309 (58)
Ex-smoker	151 (28)
Active smoker	74 (14)
Diabetes, n (%)	
No	486 (91%)
Yes	50 (9%)
Previous radiation, n (%)	
Yes	133 (25)
No	403 (75)

SD, standard deviation.

**Table 2.** Free Flap Characteristics.

Reconstructive surgery characteristics	
Number of flaps	795
Reconstruction type, n (%)	
Breast	377 (68%)
Bilateral	237 (63%)
Unilateral	140 (37%)
HN	71 (13%)
LE	88 (16%)
UE	13 (2%)
Abdominal wall	3 (1%)
Reconstructive timing, n (%)	
Delayed reconstruction	166 (30%)
Immediate reconstruction	386 (70%)
Complications, n (%)	
No	422 (76%)
Yes	131 (24%)
Flap revisions, n (%)*	
Revision of another surgeon flap	5 (1%)
Primary surgeon revision	112 (20%)
Flap failure (excluding other surgeons’ flaps)	54 (7%)
Successful salvage	28 (4%)
Unsuccessful salvage	26 (4%)

HN, head and neck; LE, lower extremity; UE, upper extremity. *Non-cosmetic revision.

### Reconstructive Surgery Characteristics

**Figure 1.**
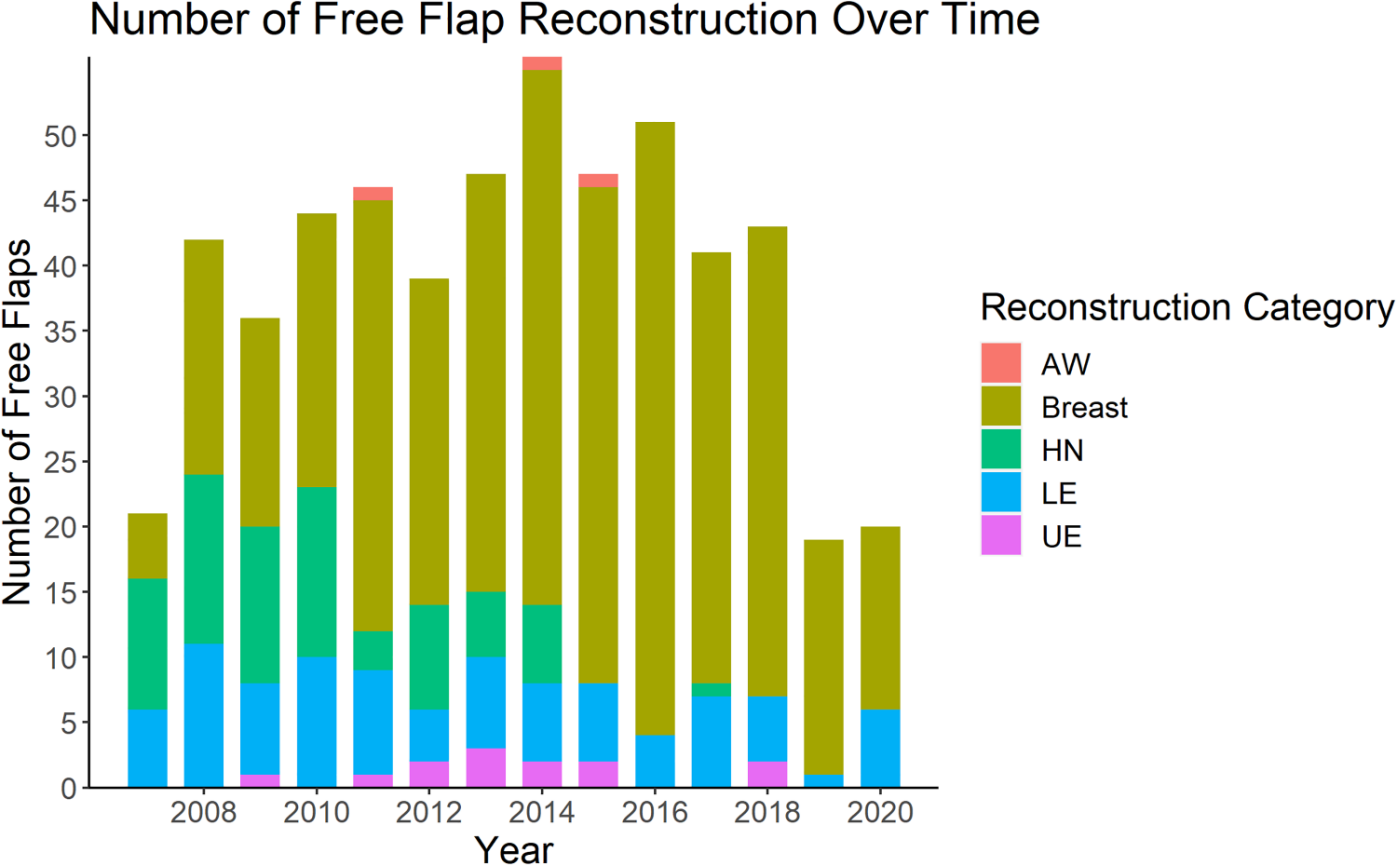
Number of free flap reconstruction over 13-year career of a single microsurgeon. AW, abdominal wall; HN, head and neck; LE, lower extremity; UE, upper extremity.

**Figure 2.**
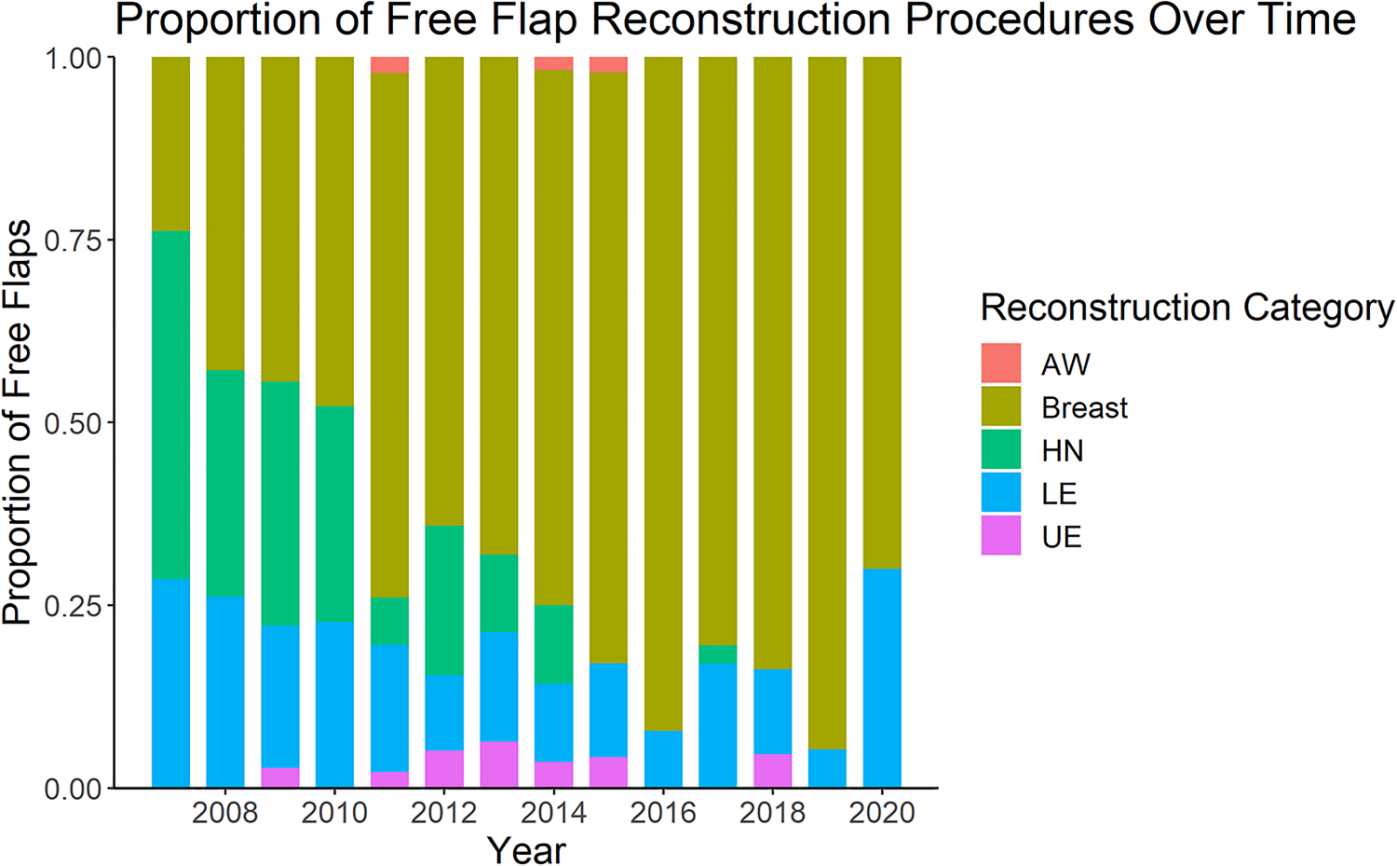
Proportion of free flap reconstruction by reconstruction category per year over career of a single microsurgeon. AW, abdominal wall; HN, head and neck; LE, lower extremity; UE, upper extremity.

The number of reconstructive procedures performed each year is shown in Figure 1. The majority of cases were breast reconstruction (377/552, 68%), of which 63% (237/377) were bilateral. Lower extremity and head and neck reconstruction made up approximately 30% of the remaining reconstructive procedures, with upper extremity and abdominal wall being the least common type of reconstruction (2% and 1%, respectively) (Table 2). Over time, there was a shift to predominantly breast reconstruction (Figure 2). There was a statistically significant association between the type of procedure performed and the year of practice (*P* < 0.005) (Figure 2). With respect to volume and diversity of cases, there was a peak in both volume and clinical diversity at years seven to nine in practice. A decline in years 12 and 13 in both categories coincided with the addition of a microsurgical partner.

### Complications

There were 131 postoperative complications following 795 free flap reconstructions. The most common complication was partial necrosis (24/795, 3.0%), followed by venous congestion (24/795, 3.0%) and postoperative infections (7/795, 0.9%). There were 10/795 cases (1.3%) of isolated arterial insufficiency, 13/795 (1.6%) venous and arterial insufficiency (13/795, 1.6%), and 11/795 (1.4%) hematoma. There were 7/795 (0.9%) seromas requiring drainage, 6/795 (0.8%) incidences of dehiscence, and 5/795 (0.6%) DVT/PE. Of the abdominal-based flaps, there were 11/72 (15.3%) abdominal wall hernias (Appendix B).

**Figure 3.**
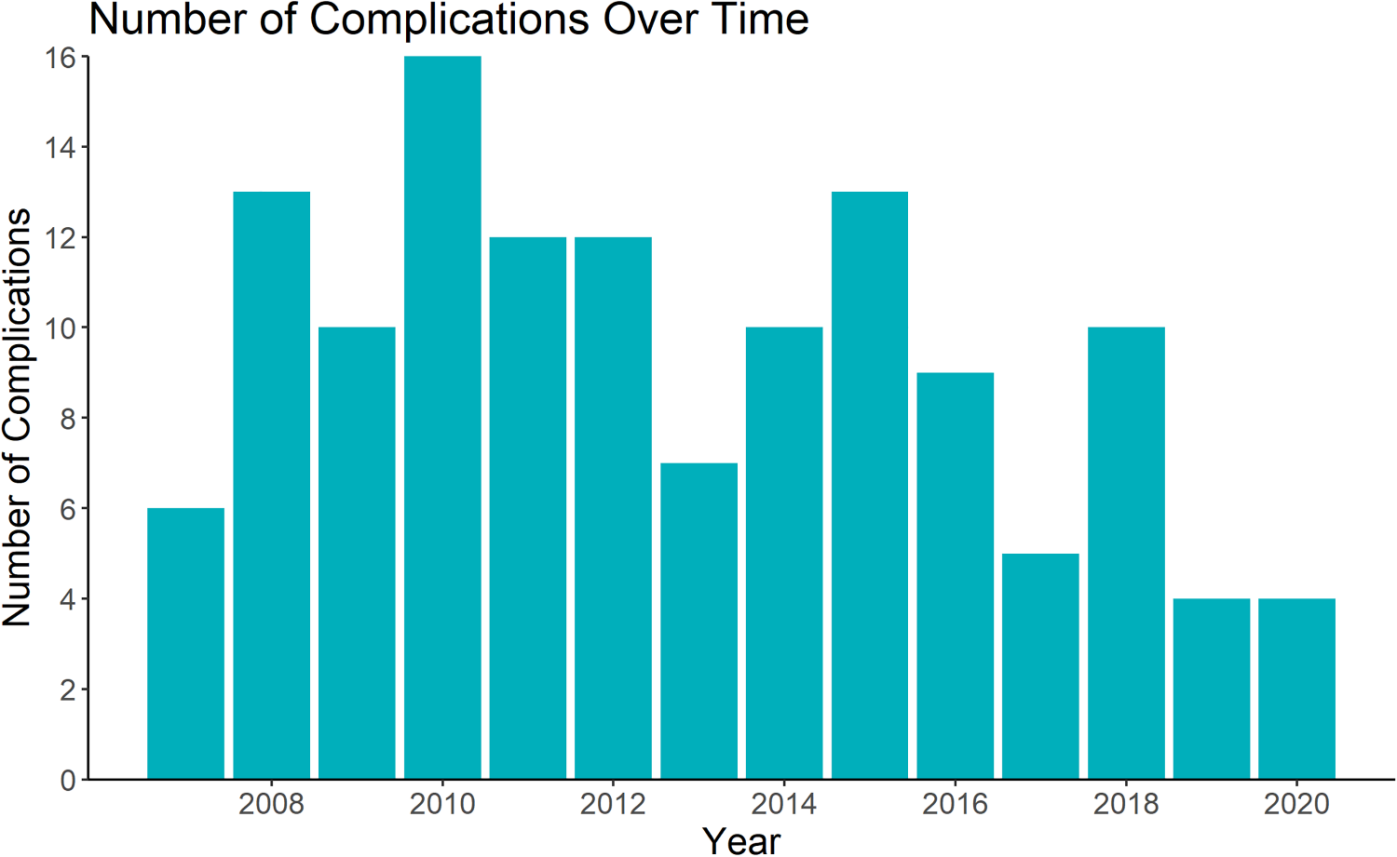
Number of free flap complications over 13-year career of a single microsurgeon.

The number of complications over time is presented in Figure 3. In unadjusted analysis, there is decreased odds of complications with each increasing year of practice (OR = 0.93, *P* < 0.05, 95% CI 0.88 to 0.99). After adjusting for the number of free flaps performed and the type of reconstruction performed this relationship was no longer statistically significant (aOR = 0.96, *P* = 0.20, 95% CI 0.90 to 1.02).

There were 54/795 (7%) flap re-explorations. Of these, 28 were salvaged, yielding an overall success rate of 96.7%. Of the 26 flap failures, 24 were fasiocutaneous or myocutaneous flaps and two were osteocutaneous flaps. There was no association between the number of years in practice and odds of flap failure (OR = 1.0, *P* = 0.99, 95% CI 0.90 to 1.11). After adjusting for the volume and type of reconstructions, this relationship remained both clinically and statistically insignificant (aOR = 1.05, *P* = 0.40, 95% CI 0.93 to 1.19).

## Discussion

In this study, we illustrate the characteristics and complications of all microvascular free tissue transfers of a single microsurgeon at an academic tertiary care center over 13 years of a broad practice; this represents approximately the first half of their career. Over time, the type of reconstructive cases performed changed, with a shift from a broad practice to primarily breast reconstruction. There was no significant change in the rate of complications with years in practice after adjusting for potential confounding factors. Overall, there were 26 flap failures with an overall success rate of 96.7%. This is comparable to previous reports in the literature.^10–12^

The practice of an academic plastic surgeon is influenced by expertise, personal interests, burden of plastic surgery-related disease, and the availability of plastic surgeons to provide care within a catchment area.^13^ In Canada and the United States, shortages of plastic surgeons have increased the workload and practice profiles in all areas of plastic surgery.^14,15^ In a 2007 survey distributed to all members of the Canadian Society of Plastic Surgeons, over 70% agreed that there was an inadequate number of plastic surgeons to fulfill the need for plastic surgery services.^14^ In the present review, the surgeon conducted the majority of reconstructive cases at the center between 2007 and 2013, with free tissue transfers distributed across head and neck, breast, abdominal wall, and extremity reconstruction (oncologic and traumatic). These gaps were filled by the hiring of four academic surgeons between 2015 and 2018. With the addition of these new staff, there was a significant change in the type of free tissue transfer performed as it permitted further sub-specialization in their area of interest. Furthermore, there has been a recent increase in the otolaryngologists trained in microvascular free tissue transfer who perform their own complex head and neck reconstruction, which also contributes to the practice shift observed in head and neck flaps.^16,17^ In addition to the type of reconstruction performed, there was a notable change in the volume of free tissue transfers in the last 2 years of data capture. The hiring of a practice partner with a comprehensive microsurgery fellowship working at the same institution in 2018 likely had the greatest impact on case load. There was a drastic decrease in the volume of elective free flap reconstruction cases conducted in 2019 which then extended into 2020 during the COVID-19 pandemic. The pandemic has had a negative impact on surgical volumes worldwide across all subspecialties. Our analyses ended in 2020 given inconsistent hospital resources.

Free flap failure and other complications of free tissue transfers are influenced by patient factors, flap-specific factors, and surgeon experience.^1^ In our study, there was no relationship between the surgeon experience and the rate of flap failure. Mahmoudi et al previously showed a positive relationship between years in practice and success of operation. This was a small association (OR 1.04, *P* < 0.001, CI 1.02–1.06) in a much larger review of 25 327 patients relative to our cohort of 552.^5^ In our cohort, the overall complication rate was 23.7%, which is comparable to previous reports.^1,18^ Based on our analyses, after controlling for the number of cases and type of reconstructions performed, there was no relationship between the odds of complications and additional years in practice in this single surgeon experience. This is likely attributable to the low sample size and retrospective nature of the study. However, the experience gained over time from completing residency and fellowship at institutions with clinically high volumes of free flap reconstruction likely contributed to efficiency, expertise, and thus, a profile with low complications from the beginning of practice.

By analyzing consecutive patients over a 13-year period, we are able to demonstrate the true complication profile of both low- and high-risk patients within one's practice. There has been a recent shift in plastic surgery literature to encourage reporting of consecutive cases to help delineate free flap complications and identify methods to reduce the risk of complication. At our institution, the majority of indications for reconstruction were malignancy (head and neck, breast, sarcoma) and soft tissue or bony defects resulting from traumatic or chronic infections. While many patient risk factors have been previously documented, optimizing conditions at an academic center among free flap reconstructions in the emergent setting is often difficult.^11,12^ New reconstructive techniques involving microvascular tissue transfer and bone graft have allowed attempts at limb salvage in the acute setting.^19,20^ In these cases, the assessment of soft tissue viability, ischemia time, bone condition, and nerve injury remain pivotal in guiding decision for reconstruction. However, we cannot control for patient comorbidities or poor injury patterns. In oncologic patients, risk of venous thromboembolism is inherently elevated due to the nature of the disease and prolonged immobilization from surgery and hospitalization.^21^ Overall, surgeons often need to take on the burden of patient comorbidities that can compromise flap viability. Thus, by analyzing consecutive free tissue transfers, we demonstrate a thorough review of the 13-year experience of a Canadian microvascular surgeon, including these high-risk scenarios.

The primary strength of this study is the inclusion of consecutive cases, providing a complete overview of case load and complication profile over one's career. This study design allows for an in-depth analysis of an individual's career, and how it has been impacted by years in practice. Previous studies have conducted large retrospective reviews of whole institutions which provide valuable information regarding overall volume, rate of flap failure, and risk factors for complications, but limit longitudinal assessment of individual surgeon practice changes.^3^ There are several limitations to our study. Firstly, we only reported outcomes on patients who received free flap reconstruction. Therefore, we were unable to evaluate the shift to other types of procedures, limiting our ability to analyze the entirety of their career. Secondly, this study is limited by variability in length of follow-up. There was no standardized follow-up period after discharge from hospital and therefore any complications that occurred beyond the latest follow-up appointment would have been missed. Thirdly, our study is limited by the sample size. Previous studies have shown a very low effect size requiring a large sample (>10 000 cases) to detect a significant change in complications over time.^4,5^ Therefore, we are unable to extrapolate changes in complication rate with years in practice to whole institutions or geographical regions. Lastly, this is a retrospective study and may be prone to inherent limitations to study design including observer bias and data entry error.

## Conclusion

In this retrospective review, we demonstrate a high free tissue transfer success rate for broad reconstruction of traumatic injuries and oncologic resections across a 13-year period in a single microsurgeon's career. The type and volume of free flap reconstruction conducted by the surgeon has changed with the addition of new plastic surgeons. In the present study, surgical experience did not have a significant impact on free flap complications, although this was limited by a small sample size. Future studies should review postoperative outcomes of free tissue transfers across multiple centers nationwide using provisional or national databases. We plan to analyze the practice pattern of additional microsurgeons in our group and extended network to highlight changes in practice patterns and postoperative complications. At our center, free tissue transfer remains a valuable tool for reconstruction in both low- and high-risk patients.

## Supplemental Material

sj-docx-1-psg-10.1177_22925503241234934 - Supplemental material for Consecutive Microsurgical Cases Performed by Single Surgeon at a Canadian Tertiary Care Center: A Retrospective Review Supplemental material, sj-docx-1-psg-10.1177_22925503241234934 for Consecutive Microsurgical Cases Performed by Single Surgeon at a Canadian Tertiary Care Center: A Retrospective Review by Andrew T. Chen, Jessica Gormley, Oluwatobi Olaiya, Sophocles H. Voineskos, Christopher J. Coroneos and Ronen Avram in Plastic Surgery
