# Supplementary material for: Consecutive Microsurgical Cases Performed by Single Surgeon at a Canadian Tertiary Care Center: A Retrospective Review
Source: Plast Surg (Oakv). 2024 Mar 1;33(3):435–41. doi: 10.1177/22925503241234934 (PMC11561944; doi:10.1177/22925503241234934)
Supplement: sj-docx-1-psg-10.1177_22925503241234934 - Supplemental material for Consecutive Microsurgical Cases Performed by Single Surgeon at a Canadian Tertiary Care Center: A Retrospective Review [file sj-docx-1-psg-10.1177_22925503241234934.docx]

**Appendix:**

**Appendix A.** OHIP Billing Codes

| **Code** |  |
| --- | --- |
| R064 | Elevation of free island skin and subcutaneous flap and closure of defect |
| R065 | Preparation of microvascular recipient site for free island skin subcutaneous flap |
| R066 | Transplantation of free island skin and subcutaneous flap with microvascular anastomosis(es) |
| R129 | Preparation of microvascular recipient site for muscle, tendon and nerve anastomosis(es) |
| R135 | Preparation of microvascular recipient site for free island skin and bone flap |

**Appendix B.** Complication Rates by Category

| **Complications** | **N (%)** |
| --- | --- |
| Partial necrosis | 24 (3.0) |
| Venous congestion | 24 (3.0) |
| Infection | 16 (2.0) |
| Venous congestion and arterial insufficiency | 13 (1.6) |
| Hematoma | 11 (1.4) |
| Abdominal hernia | 11 (1.4) |
| Arterial insufficiency | 10 (1.3) |
| Seroma | 7 (0.9) |
| Wound dehiscence | 6 (0.8) |
| DVT/PE | 5 (0.6) |
| Failure unknown | 2 (0.3) |
| Myocardial infarction | 1 (0.1) |
| Death | 1 (0.1) |
